# Impact Resistance Analysis and Optimization of Variant Truss Beam Structure Based on Material Properties

**DOI:** 10.3390/ma14195847

**Published:** 2021-10-06

**Authors:** Xiaohao Li, Junqi Pan, Xingchen Zhou

**Affiliations:** 1 School of Mechanical Engineering and Automation, Northeastern University, Shenyang 110819, China; chn202003_sy@163.com (J.P.); neuzstu2019@yeah.net (X.Z.); 2 Key Laboratory of Vibration and Control of Aero-Propulsion Systems Ministry of Education of China, Northeastern University, Shenyang 110819, China

**Keywords:** variant truss beam, impact resistance analysis, structure optimization, material properties

## Abstract

In order to meet the increasing application requirements with regards to structural impact resistance in industries such as mining, construction, aerospace engineering, and disaster relief and mitigation, this paper designs a variant truss beam structure with a large shrinkage ratio and high impact resistance. Based on the principle of the curved trajectory of scissor mechanisms, this paper conducts a finite element simulation analysis of the impact load on the truss beam structure, a theoretical analysis of the impact response and a relevant prototype bench-top experiment, completing a full study on the impact resistance mechanism of the designed variant truss beam structure under the impact load. In the paper, the buffer effect of the external load impact on the variant truss beam structure is analyzed from the perspective of the energy change of elastic–plastic deformation. This paper proposes an optimization strategy for the variant truss beam structure with the energy absorption rate as the optimization index through extensive analysis of the parameter response surfaces. The strategy integrates analyses on the response characteristic analysis of various configuration materials to obtain an optimal combination of component parameters that ensures that the strength of the truss beam structure meets set requirements. The strategy provides a feasible method with which to verify the effectiveness and impact resistance of a variant truss structure design.

## 1. Introduction

At present, the scissor-type truss beam mechanism has been widely used in mining, building construction, aerospace development projects, emergency rescue and disaster reduction and other industries [1,2,3,4,5]. This mechanism has the characteristics of large shrinkage ratio and structural compliance rotation. It can bear the impact of an external load well, and has the characteristics of small storage volume, small mass and good impact resistance performance.

Many scholars have conducted in-depth studies on the design and movement mechanism of truss structures [6,7,8,9,10,11]. Based on the design of the ring-flat-head cone truss structure with its deployable mechanism [12], the evolution law and movement characteristics of the variant scissor mechanism has been analyzed and a more comprehensive discussion surrounding the degree of freedom of its motion has been carried out [13]. Based on the elastic finite element, the deformation mode and stress-strain distribution of the truss structure were discussed, and the relationship between truss deformation and impact force was studied through regression analysis [14]. Due to the complexity of the variant scissor mechanism structure and its multiple topologies, researchers conducted an in-depth discussion on the topology of the truss mechanism from multiple angles [15,16,17,18,19]. For example, Li et al. [15] analyzed the multi-topological, structural characteristics of the space truss unfolding mechanism, and proposed a mechanism topology analysis method based on the metamorphic principle; Huang et al. [16] used the Jordan Speed Variation Principle to model the dynamics of the flexible components of the variable topological space deployable truss and analyzed the law of impact change on the truss unfolding motion. At present, researchers have also carried out a number of studies on the mechanical properties of truss structures [20,21,22,23]. For example, Sun et al. [20] deeply studied the vibration control of scissor truss and pointed out that the scissor structure had a good load-bearing capacity and balance stability. However, this research method is not ideal for solving the problem of sudden impact load. Li et al. [22] put forward an analysis method for the impact load stability of ring truss structures by modal analysis of the truss model, but practical application showed that this method had great limitations for the analysis of other truss shapes. Although much of the literature is related to truss structure research, there are few research results on the design of variant truss beam structures, mechanical response characteristics under impact loads, impact resistance mechanisms and structure optimization.

In this paper, by adjusting the position of the middle pin in the traditional scissor unit, the variant structural unit of the unequal-length scissor arm was obtained, and the multiple variant structural units are operated in series and in parallel to obtain the variant truss beam structure. Through theoretical derivation, finite element dynamics simulation and physical experiment, this paper reveals the mechanical response characteristics and deformation mechanism of the variable truss beam structure element under impact load. Taking specific energy absorption [24,25] as an index, the parameter response surface optimization method for the truss beam structure was studied, and the impact resistance of the structure was optimized within a certain mass range.

## 2. Design of Variant Truss Beam Structure

**Figure 1:**
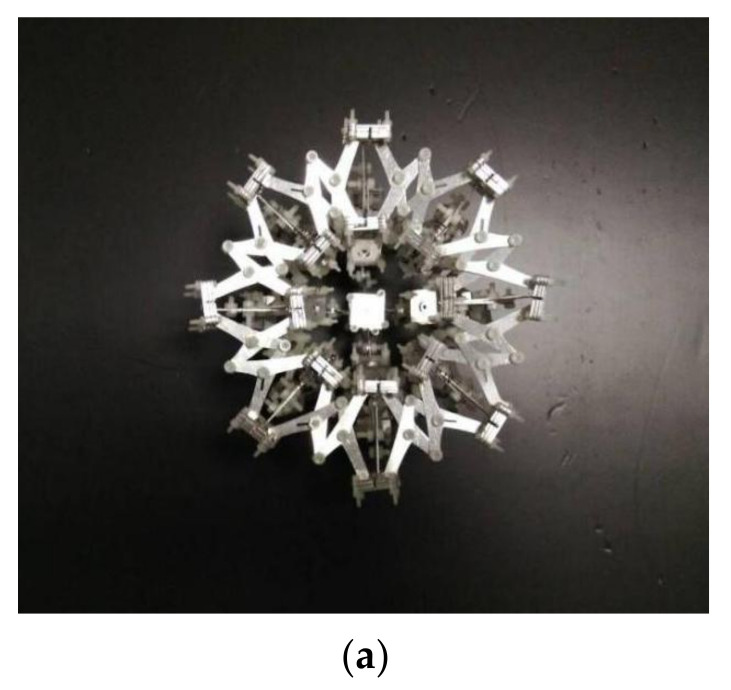

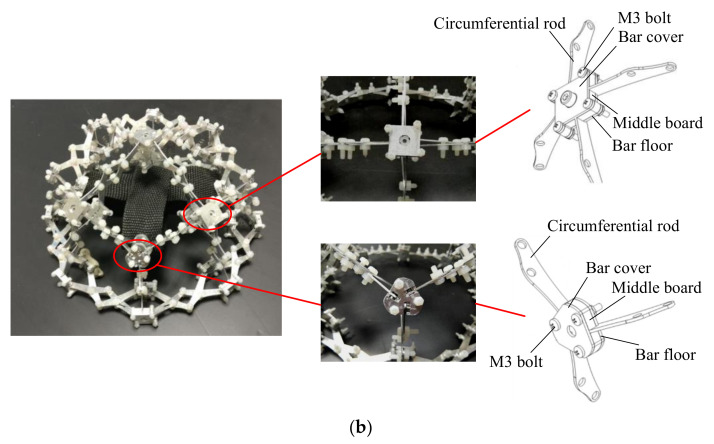
Variant truss beam structure: (**a**) Contracted state of the truss beam structure; (**b**) Expanded state of the truss beam structure and constituent elements.

In view of the user requirements that the mass of the truss beam structure is as small as possible, the impact resistance is as large as possible, and the change rate of the structure’s internal cavity volume is as small as possible when it fails, we designed the variable shrinkage type truss beam structure as shown in Figure 1 (Figure 1a shows the contracted state of the truss beam structure, and Figure 1b shows the expanded state of the truss beam structure). The truss beam structure is composed of basic units such as 4-claw joints and 3-claw joints, as shown in Figure 1b, which are composed of multiple sets of series and parallel connections. The structure has the characteristics of a large contraction ratio and small storage space, and a single constraint in any direction of the truss beam structure can limit the contraction and expansion movement of the mechanism. To its advantage, the structure has a single degree of freedom and stable movement.

Through the impact dynamic response analysis and the structural parameter optimization of the truss beam structure, it shows excellent impact resistance within a certain mass range.

## 3. Analysis of the Anti-Impact Dynamics Mechanism of Variant Truss Beam Structure

### 3.1. Finite Element Simulation Analysis of Impact Load of Truss Beam Structural Element

**Figure 2:**
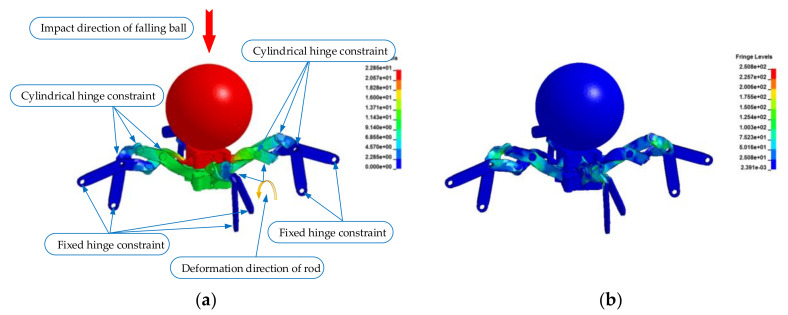
Simulation of the truss beam element under impact load: (**a**) Plastic deformation of the element; (**b**) Equivalent stress distribution of the element.

In order to quantitatively analyze the stress distribution and strain of the truss structure element after impact, the three-dimensional model of the truss structure element was established using SolidWorks software, which was imported into ANSYS software for finite element dynamic analysis, and the stress characteristics and deformation mechanism of the structural element were simulated. Among them, the beam joints and the O-joint were restrained by cylindrical hinges, and the beam members and supports were restrained by fixed hinges (the constraint type and location are indicated in Figure 2 of this paper).

This calculation example uses 2.0–4.5 aluminum truss beam members (that is, the thickness of the member is 2.0 mm, and the width is 4.5 mm). The material parameters of each component of the truss beam structural unit are shown in Table 1 (the data in Table 1 are quoted from [26]).

**Figure 3:**
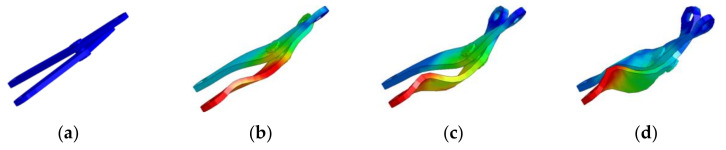
Deformation process of truss beam structure members under impact load: (**a**) Shape of the rod in the initial state; (**b**) Process of elastic deformation of the rod just after the impact; (**c**) Shape of the rod in contact with the rod groove in the intermediate transition process; (**d**) Final shape in the simulation analysis of the rod.

The experimental impact load was a steel falling ball with a weight of 380.1 g and the impact acceleration was 9805.3$\;\mathrm{mm}/s^{2}$, while the instantaneous velocity of the impact was 4996.1 mm/s. Figure 2a is the plastic deformation of the truss beam structural element under the impact load, Figure 2b is the equivalent stress distribution of the impact load, and Figure 3 is the deformation process of the rod after the impact.

Figure 3b shows that the force on the rod at this point is mainly the force between the pin and the hole. The O-shaped joint rotates in the clockwise direction, and the deformation of the rod is elastic deformation. In Figure 3c, the rod is simultaneously acted by the pin shaft and the rod groove, the O-joint rotates counterclockwise, and the rod deforms in the opposite direction. At this point, the rod gradually transforms from elastic deformation to plastic deformation. As demonstrated by Figure 3d, the main deformation of the final rod is plastic deformation, showing a wrinkled form. This is caused by the rotation of the O-joint in two directions before and after, and the change of the force onto the rod.

Through a large number of impact load simulation experiments, the impact mechanics characteristics of the truss beam structure were obtained: (1) Under the condition of an impulse of 1,858,500 g·mm/s, the truss beam structure will lose stability, and the members will quickly reach the stage of plastic deformation. (2) Under the impact load, the stress distribution on the truss is very uneven, and the absorption efficiency of the impact energy is not good.

### 3.2. Theoretical Analysis of Impact Load of Truss Structure Elements

1. Initial phase

**Figure 4:**
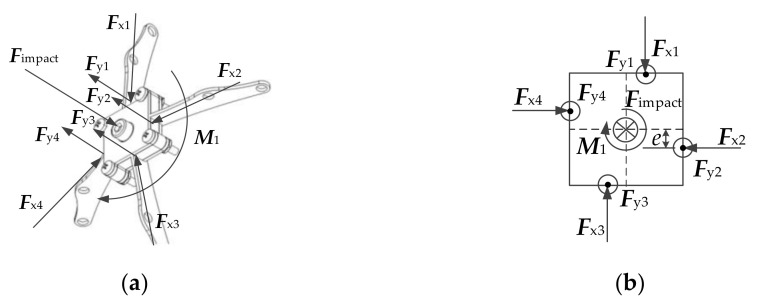
Force analysis of O-joint in initial stage: (**a**) Force distribution diagram; (**b**) Force analysis diagram.

In the initial stage, the rod was located in the center of the rod groove, without touching the rod groove. The force acting on the O-joint is shown in Figure 4.

It is known from Figure 4 that:

(1) $$F=\overset{4}{\underset{i=1}{\sum }}F_{yi}$$

where, *F* is the impact and $F_{yi}$ is the component of force in the vertical direction toward the O-joint.

At this stage, the force following the rod resisted against the impact. At the same time, the O-joint twisted, resulting in changes in angle:

(2) $$dM=\overset{2}{\underset{j=1}{\sum }}dM_{j}$$

(3) $$dM_{1}=M_{13}=e\left(dF_{x1}+dF_{x3}\right)$$

(4) $$dM_{2}=M_{24}=e\left(dF_{x2}+dF_{x4}\right)$$

where, M is the force couple that the horizontal component of the force acts on the O-joint; $M_{1}\;$ is the force couple produced by $F_{x1}$ and $F_{x3}$; $M_{2}\;$ is the force couple produced by $F_{x2}$ and $F_{x4}$; e is the horizontal moment arm length of the force applied to the O-joint; $F_{xi}$ is the horizontal component of the force toward the O-joint.

Consider the balance of forces in Figure 4, then $\left|F_{x1}\right|=\left|F_{x2}\right|=\left|F_{x3}\right|=\left|F_{x4}\right|$.

Substituting Equations (3) and (4) into Equation (2), we arrive at

(5) $$dM=4edF_{x1}$$

Based on Newton’s Second Law of Motion-Force and Acceleration and Equation (5), the torsional angular acceleration produced by the O-joint under M is:

(6) $$d\alpha =\frac{dM}{J}$$

where, *α* is angular acceleration of O-joint; J is the moment of inertia of O-joint.

Based on Equations (5) and (6), the angle Δθ that O-joint turns is:

(7) $$\Delta \theta =\iint d\alpha \mathrm{dt}=\iint \frac{4edF_{x1}}{J}\mathrm{dt}$$

2. Intermediate transition state

**Figure 5:**
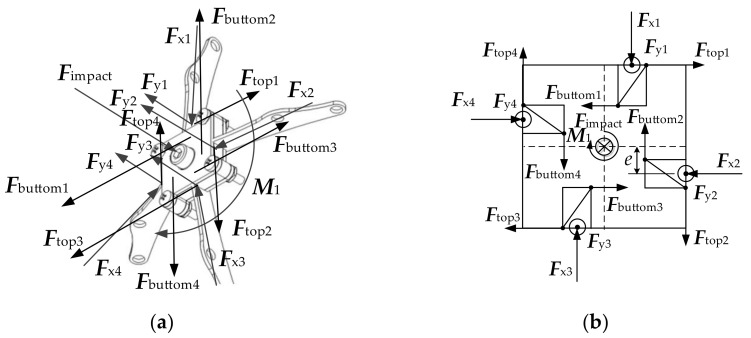
Force analysis of O-joint in intermediate transition state: (**a**) Force distribution diagram; (**b**) Force analysis diagram.

A force generated by the contact between the rod and rod grooves caused by the torsion of O-joint. Figure 5 shows the force analysis:

(8) $$dM_{\mathrm{buttom}}=\overset{2}{\underset{j=1}{\sum }}dM_{\mathrm{buttom}\;j}$$

where,

(9) $$dM_{\mathrm{buttom}\;1}=dM_{\mathrm{buttom}\;13}=e\left(dF_{\mathrm{buttom}\;1}+dF_{\mathrm{buttom}\;3}\right)$$

(10) $$dM_{\mathrm{buttom}\;2}=dM_{\mathrm{buttom}\;24}=e\left(dF_{\mathrm{buttom}\;2}+dF_{\mathrm{buttom}\;4}\right)\;$$

and $M_{\mathrm{buttom}}$ is the total force couple generated by the force applied to the O-joint at the contact point of the rods with the groove bottom; $M_{\mathrm{buttom}\;1}\;$ is the force couple generated by the force applied to the O-joint at the contact point of the first rod and the third rod with the groove bottom; $M_{\mathrm{buttom}\;2}$ is the force couple generated by the force applied to the O-joint at the contact point of the second rod and the fourth rod with the groove bottom; $F_{\mathrm{buttom}\;1}$, $F_{\mathrm{buttom}\;2}$, $F_{\mathrm{buttom}\;3}$ and $F_{\mathrm{buttom}\;4}$ is the force applied to the O-joint at the contact point of the first rod, the second rod, the third rod and the fourth rod with the groove bottom.

Consider the balance of forces in Figure 5, then $\left|F_{\mathrm{buttom}\;1}\right|=\left|F_{\mathrm{buttom}\;2}\right|=\left|F_{\mathrm{buttom}\;3}\right|=\left|F_{\mathrm{buttom}\;4}\right|$.

Substituting Equations (9) and (10) into Equation (8), we obtain

(11) $$dM_{\mathrm{buttom}}=4edF_{\mathrm{buttom}\;1}$$

In the same way, we obtain,

(12) $$dM_{\mathrm{top}}=\overset{2}{\underset{j=1}{\sum }}dM_{\mathrm{top}\;j}$$

(13) $$dM_{\mathrm{top}\;1}=dM_{\mathrm{top}\;13}=e\left(dF_{\mathrm{top}\;1}+dF_{\mathrm{top}\;3}\right)$$

(14) $$dM_{\mathrm{top}\;2}=dM_{\mathrm{top}\;24}=e\left(dF_{\mathrm{top}\;2}+dF_{\mathrm{top}\;4}\right)$$

where, $M_{\mathrm{top}}$ is the total force couple generated by the force applied to the O-joint at the contact point of the rods with the top of the groove; $M_{\mathrm{top}\;1}$ is the force couple generated by the force applied to the O-joint at the contact point of the first rod and the third rod with the top of the groove; $M_{\mathrm{top}\;2}$ is the force couple generated by the force applied to the O-joint at the contact point of the second rod and the fourth rod with the top of the groove; $F_{\mathrm{top}\;1}$, $F_{\mathrm{top}\;2}$, $F_{\mathrm{top}\;3}$ and $F_{\mathrm{top}\;4}$ is the force applied to the O-joint at the contact point of the first rod, the second rod, the third rod and the fourth rod with the top of the groove.

Consider the balance of forces in Figure 5, then $\left|F_{\mathrm{top}\;1}\right|=\left|F_{\mathrm{top}\;2}\right|=\left|F_{\mathrm{top}\;3}\right|=\left|F_{\mathrm{top}\;4}\right|$.

Substituting Equations (13) and (14) into Equation (12), we obtain

(15) $$dM_{\mathrm{top}}=4edF_{\mathrm{top}\;1}$$

Here, the total force couple applied to the O-joint is

(16) $$dM=\overset{2}{\underset{j=1}{\sum }}dM_{j}+\overset{2}{\underset{j=1}{\sum }}dM_{\mathrm{top}\;j}-\overset{2}{\underset{j=1}{\sum }}dM_{\mathrm{buttom}\;j}$$

Substitute Equations (2), (11) and (15) into Equation (16), we obtain:

(17) $$dM=4e\left(dF_{x1}+dF_{\mathrm{top}\;1}-dF_{\mathrm{buttom}\;1}\right)$$

Then put Equation (17) into Equation (7), the angle that the O-joint turns shown as Equation (18) is:

(18) $$\Delta \theta =\iint \frac{2e\left(dF_{x1}+dF_{\mathrm{top}\;1}-dF_{\mathrm{bottom}\;1}\right)}{J}\mathrm{dt}$$

If the minimal deformation effect of the rod in the end can be ignored, when the O-joint turned angle Δθ, expressed as Equation (18), the amount of change in the length of the rod Δ*l* can be approximated as Equation (19):

(19) $$\Delta l=l\times \left(1-\cos\left(\Delta \theta \right)\right)$$

where, Δl is the length of the rod changed; l is the original length of the rod.

Based on Equations (18) and (19), the distance Δh of the O-joint descending in the vertical direction can be approximated as:

(20) $$\Delta h=\frac{\Delta l}{\sin\beta }$$

where, Δh is the distance that the O-joint descends in the vertical direction, which can be used to characterize the displacement of the truss beam structure shown in Figure 1 along the direction of the impact force. The value of Δh. can be used to reflect the ability of the truss beam structure we designed (as shown in Figure 1) to resist the deformation of the impact load. β is the angle that the rod changes in the vertical direction when the O-joint produces the displacement Δh in the vertical direction, which can be obtained by measurement.

In conclusion, the impact resistance was generated through components of the force toward the O-joint in the vertical direction which decreased with the increase in displacement of the O-joint in the vertical direction. More importantly, the O-joint was rotated to cushion the impact loads, reducing the impacts on the whole structure when undergoing them, while the rod absorbed the impact of the load energy through the deformation. This proves the validity and rationality of the truss beam structure design shown in Figure 1.

### 3.3. Physical Experiment of Impact Load of Truss Beam Structure

**Figure 6:**
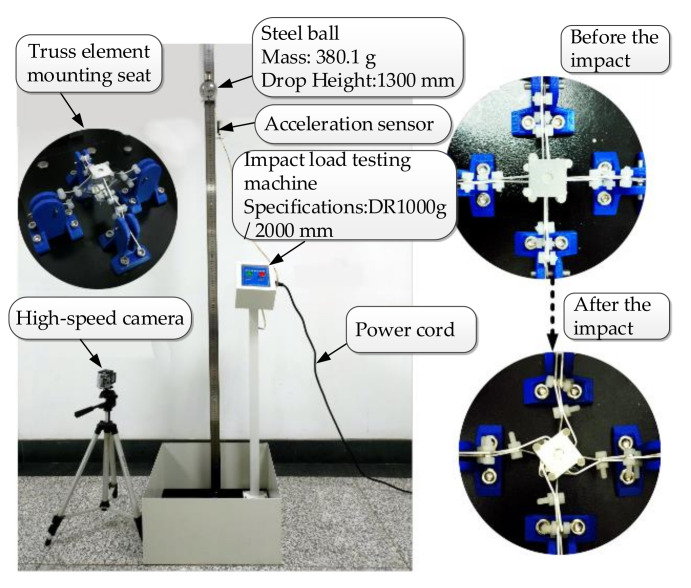
Experimental device and impact test.

A DR1000 g/2000 mm falling-ball impact tester was used, with 380.1 g steel balls and the falling-ball height set to 1300 mm. The truss beam structure was firmly connected with the lower base of the testing machine using PLA material brackets. The experimental layout is shown in Figure 6.

The physical experiment adopted the same model and experimental conditions as the simulation experiment. The steel ball with an impulse of 1,858,500 $g\cdot \mathrm{mm}/s^{2}$ impacts the structural elements of the composite truss beam with different widths and thicknesses. The real deformation process of the rod was recorded quantitatively using a high-speed camera. The plastic deformation or elastic deformation of the joint element impact load was analyzed, and the buffer mechanism of the truss beam structure to the impact load was explored from the perspective of energy change.

**Figure 7:**
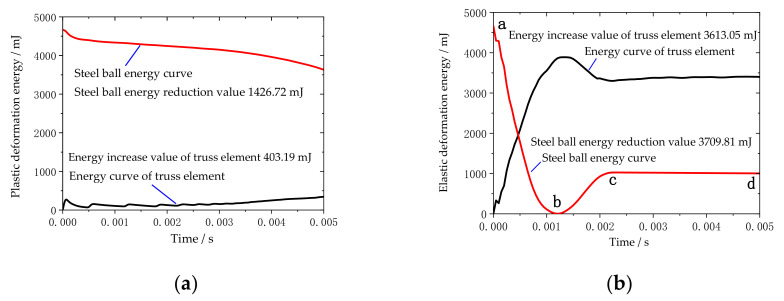
Law of energy change of truss beam structure: (**a**) Energy change under plastic deformation; (**b**) Energy change under elastic deformation.

Take the variable truss beam structural unit composed of 1.5–3.0 aluminum rods as the experimental object. According to the high-speed camera, shooting at a frame rate of 240FPS, the impact process between the steel ball and the structural unit is extremely short, and the O-joint is twisted clockwise. The parts are shown in Figure 3b,c, with obvious plastic deformation and weak elastic deformation, which is the same as the simulation result. Figure 7a demonstrates the experimental data analysis of the energy changes in the truss beam structure and the steel ball over time. In this case, the truss beam structure element mainly undergoes plastic deformation of the rods, thus the energy absorption characteristic of the truss beam structure slowly increases over time and tends to be stable, mainly because of the beam’s ability to absorb energy drops sharply after plastic deformation. The energy absorption efficiency of the plastically deformed rods is low [27]. At this point, the steel ball still has a large impact kinetic energy.

The variable truss beam structural unit composed of 2.0–4.5 aluminum rods is used as the experimental object. According to the high-speed camera shooting at a frame rate of 240FPS, the O-joint is first twisted clockwise, and then twisted counterclockwise. It presents an obvious fold shape as shown in Figure 3d, mainly elastic deformation. The energy changes in the truss beam structure and the steel ball over time are shown in Figure 7b. In the figure, the energy change of the steel ball is mainly divided into three stages. The ab stage is the process from the contact between the ball and the truss until the ball velocity is zero, mainly through the elastic deformation of the rod to absorb the impact energy [27]; the bc stage is the stage where the steel ball rebounds until it breaks away from the truss beam structure. As the rods do work on the steel ball, the elastic potential energy of the rods is partially converted into the kinetic energy of the rebounding steel ball. The cd stage is the ball completely separated from the truss structure. At this point, the energy of the structural unit and the steel ball tends to be stable and unchanging.

## 4. Optimization of the Rod Parameters of Variant Truss Beam Structure

The size and material of the truss rods will affect the force limit and mass of the truss beam structure. In order to optimally consider the two goals of the truss beam structure in terms of force limit and element mass, we used specific energy absorption S=E/M as the structural optimization index of the truss beam structure. In the literature, the specific energy absorption is the utilization rate of the material when the structural unit is impacted [25]. The greater the specific energy absorption, the more fully utilized the material, and the more economical the design of the structure under this condition.

We chose aluminum and steel, two commonly used metal materials, to design the width and thickness of the truss beam rods, respectively. According to the use characteristics of the variable truss beam structure [28], the thickness of the rods constituting the truss beam structure is limited to the range of 0.8 mm–3.0 mm, and the width of the rods is limited to the range of 4 mm–10 mm. Orthogonal experimental design has been carried out on the rods of the two materials in this range. Using the calculated specific energy absorption value of the impact energy in the experiment in Section 3.3 under the different design dimensions of the rods, 26 groups of evenly distributed specific energy absorption data were extracted, as shown in Table 2 and Table 3.

Comparing the experimental data in Table 2 and Table 3, the specific energy absorption of the aluminum rods is higher than that of the steel rods, mainly because the density of steel materials is 2.91 times that of aluminum.

In order to find the thickness and width of the corresponding material when the specific energy absorption S of the truss beam structure in Figure 1 is the largest, we introduced the parameter response surface method [29,30], and constructed the response surface represented by $\tilde{y}\left(x\right)$ as:

(21) $$\tilde{y}\left(x\right)=\overset{N}{\underset{j=1}{\sum }}\alpha_{j}\varphi_{j}\left(x\right)$$

where, the basis function $\varphi_{j}\left(x\right)$ is the function of the design variable $x\in E^{n}$, N is the number of terms of the selected basis function $\varphi_{j}\left(x\right)$, $\alpha_{j}$ is the adjustment parameter. For the selection of basis function $\varphi_{j}\left(x\right)$, we adopted the form of linear basis function.

According to the derivation of Chen [31], the non-linear relationship between specific energy absorption and thickness and width of a truss structure can be more accurately described in the form of a fourth-order basis function, with thickness *x* and width *y* as variables.

$E=\mathrm{RSM}\left(x,y\right)$ is the quartic basis function of thickness x and bar width y, the specific expression is

(22) $$\begin{array}{cc} E=\mathrm{RSM}\left(x,y\right)= & \alpha_{1}+\alpha_{2}x+\alpha_{3}y+\alpha_{4}x^{2}+\alpha_{5}xy+\alpha_{6}y^{2}+\alpha_{7}x^{3}+\alpha_{8}x^{2}y+\alpha_{9}xy^{2}+\alpha_{10}y^{3}+\alpha_{11}x^{4}+\alpha_{12}x^{3}y\\ & +\alpha_{13}x^{2}y^{2}+\alpha_{14}xy^{3}+\alpha_{15}y^{4} \end{array}$$

where, $\alpha ={\left[\alpha_{1},\alpha_{2},\cdots ,\alpha_{15}\right]}^{T}$ is the undetermined coefficient, the value of which is determined by nonlinear fitting based on the experimental results of finite element simulation.

Reasonably select the experimental combination of thickness and width in the design space, and perform nonlinear regression on the experimental data of specific energy absorption. Using the Levenberg–Marquardt method and the joint general global optimization method, the adjustment parameters of the aluminum material are selected under the premise of ensuring the degree of fit:

(23) $$\begin{array}{c} \alpha ={\left[\alpha_{1},\alpha_{2},\cdots ,\alpha_{15}\right]}^{T}=\left[-698.238\right.,-2047.932,\;861.290,\;1672.772,\;165.523,-203.793,-451.391,\\ -161.305,\;16.539,\;17.529,\;43.706,\;19.594,\;3.962,-1.527,{\left.-0.494\right]}^{T} \end{array}$$

**Figure 8:**
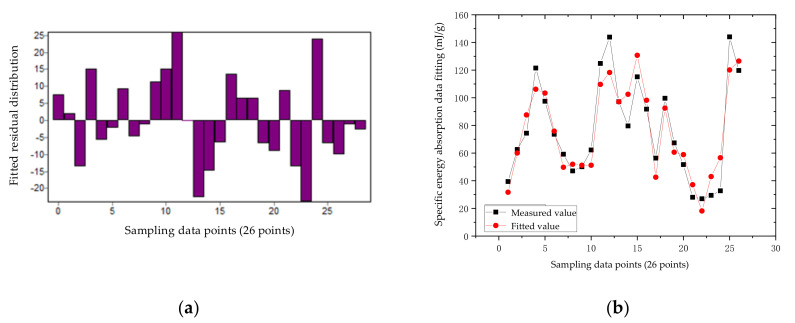
Non-linear regression statistical parameters of the aluminum truss beam structure: (**a**) Residual error distribution; (**b**) Fitting error.

According to the data in Table 2, the residual distribution and fitting error of the nonlinear regression equation of the aluminum truss beam structure can be obtained, as shown in Figure 8a,b. According to the residual distribution in Figure 8a, it can be seen that the residual points are more evenly located in the horizontal area, indicating that the selected model is more appropriate; the width of the band-shaped area is very narrow, and the maximum height is 25, which indicates that the fitting accuracy of the model is very high. From Figure 8b, we observe that the fitting error is generally very small, with an average fitting error of 2.92%. The numerical error between the response surface and the theoretical calculation is within the allowable range. The equation meets the accuracy requirements, that is, the model has an ideal fit to the response, and the model can be optimized. In the figure, the correlation coefficient (R) of the non-linear regression of aluminum material is 0.939, and the coefficient of determination (DC) is 0.882.

**Figure 9:**
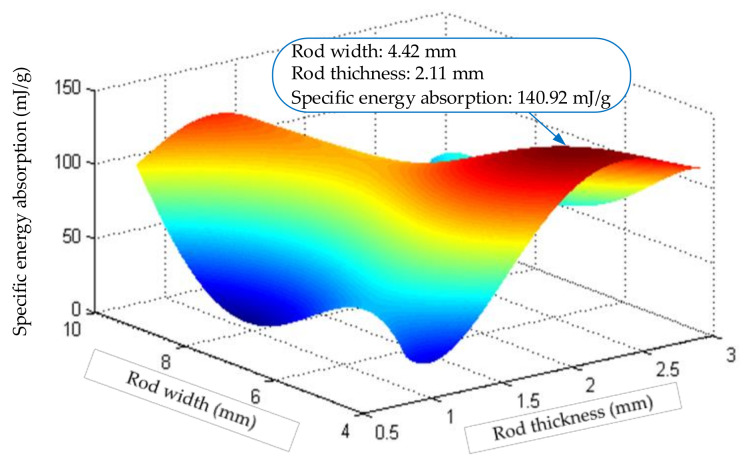
Response surface calculation diagram of Aluminum truss beam structure.

Using the obtained coefficients and equations, the response surface of the aluminum rod can be obtained as shown in Figure 9:

Figure 9 shows that when the rod is made of aluminum, when the rod thickness H = 2.11 mm and the width B  = 4.42 mm, the specific energy absorption of the structure is the largest, and the maximum specific energy absorption is 140.920 mJ/g. Through experimental verification, when the rod thickness H = 2.11 mm and width B = 4.42 mm, the actual value is 138.53 mJ/g, and the error is only 1.725%, which verifies the correctness of the response surface.

Similarly to the aluminum material analysis method, we calculated the adjustment parameters of the steel material as:

(24) $$\begin{array}{c} \alpha ={\left[\alpha_{1},\alpha_{2},\cdots ,\alpha_{15}\right]}^{T}=\left[-326.610\right.,191.295,168.739,-173.548,11.236,-38.516,71.943,\\ -9.584,\;0.597,\;3.553,-9.533,\;0.087,\;0.790,-0.175,{\left.-0.109\right]}^{T} \end{array}$$

**Figure 10:**
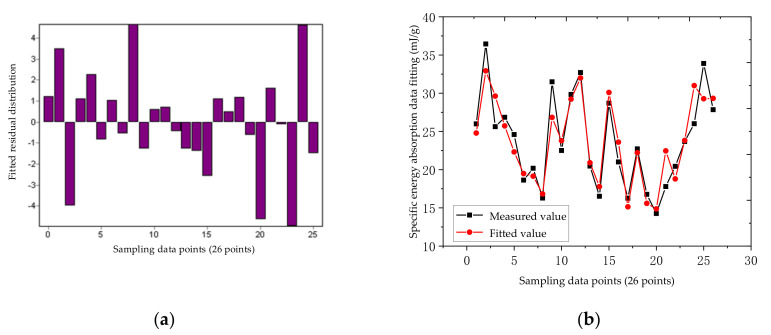
Non-linear regression statistical parameters of the steel truss beam structure: (**a**) Residual error distribution; (**b**) Fitting error.

**Figure 11:**
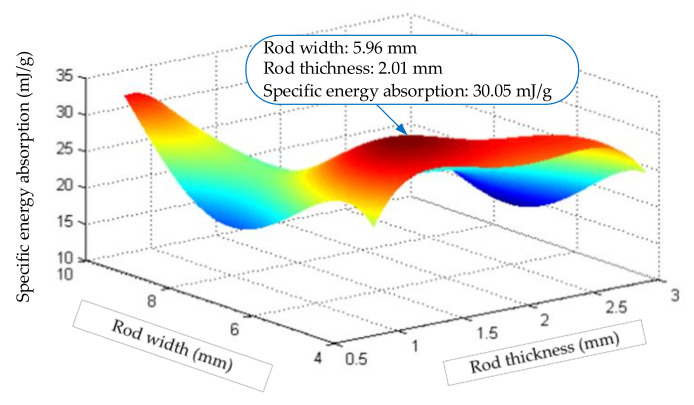
Response surface calculation diagram of Steel material.

According to the data in Table 3, the residual error distribution and fitting error of the nonlinear regression equation of the steel truss beam structure can be obtained as shown in Figure 10, and the response surface of the steel rod is shown in Figure 11.

The non-linear regression correlation coefficient (R) of the steel rods is 0.916, and the determination coefficient (DC) is 0.84. The error between the parameter response surface of the tested structure and the theoretical calculation value is 0.827%, which is within the allowable range. From the response surface as shown in Figure 11 of the steel rod, when the rod thickness H = 2.01 mm and the width B = 5.96 mm, the specific energy absorption of the structure is the largest, and the maximum is 35.051 mJ/g. Compared with the results shown in Figure 9 for the aluminum rods, the energy absorption characteristics of the steel materials were poor, mainly due to the higher density of the steel materials.

Comprehensive discussion results can be obtained, among the materials participating in the discussion, when the rod is made of aluminum material, and the specific energy absorption of the structure is the largest. That is, when the structural members of the variable truss beam are made of aluminum material, the thickness H = 2.11mm, and the width B = 4.42 mm, the designed structural unit is the most economical. As gathered from the 2.3 physical impact test (with 2.0–4.5 Aluminum rods as the object), this structural parameter can achieve a light weight under the premise of ensuring high-impact resistance.

## 5. Conclusions

In this paper, a variant truss beam structure with a large shrinkage ratio was designed, and the impact mechanism of the basic components of the structure under the impact load was analyzed, along with the deformation buffer mechanism of the components. The results show that under a certain impact load, the buffer capacity during elastic deformation is better than that during plastic deformation.

A novel optimization method is proposed for the truss structure which could meet the mechanical strength requirement for heavy impact load and minimize the amount of truss materials.

The objective of this method is to optimize the specific energy absorption and build an equation of Energy absorption-Response surface based on the parameters of the structure thickness (H) and width (B).

Then, an optimal combination of the component parameters was obtained using the Levenberg–Marquardt method and a joint global optimization method. When the truss beam structure is made of industrial aluminum material with a thickness of 2.11 mm and a width of 4.42 mm, the largest specific energy absorption of the structural elements is obtained, and the utilization rate of the material reaches the highest value.

## Figures and Tables

**Table 1:** Material parameters of the truss beam structure.

Material Properties	Steel	AL
Density/kg · m^−3^	7850	2700
Young’s modulus/MPa	200,000	6900
Poisson’s ratio	0.30	0.33
Yield strength/MPa	620.42	240
Tangent modulus/MPa	79,000	2700

**Table 2:** Aluminum material specific energy absorption data.

AL: Width(mm)/Thickness(mm)	0.8	1.1	1.4	1.7	2	2.3	2.6	2.9
3	39.411	-	32.720	-	135.055	-	119.739	-
4	-	62.618	-	136.751	-	138.018	-	99.657
5	27.403	-	74.369	-	-	-	-	-
6	-	50.105	-	121.490	-	91.766	-	67.357
7	26.966	-	-	-	97.478	-	-	-
8	-	62.227	-	97.189	-	73.638	-	51.704
9	29.448	-	-	-	-	-	59.171	-
10	-	124.812	-	79.658	-	56.362	-	47.102

**Table 3:** Steel material specific energy absorption data.

Steel: Width(mm)/Thickness(mm)	0.8	1.1	1.4	1.7	2	2.3	2.6	2.9
3	25.994	-	26.007	-	33.897	-	27.849	-
4	-	34.443	-	32.708	-	28.695	-	22.720
5	17.788	-	25.626	-	35.051	-	-	-
6	-	31.497	-	26.851	-	21.006	-	16.773
7	20.435	-	-	-	24.597	-	-	-
8	-	22.510	-	-	-	18.635	-	14.258
9	23.686	-	-	-	-	-	20.182	-
10	-	29.842	-	16.519	-	16.268	-	16.280

## Data Availability

Data sharing does not apply to this article because no new data were created in this study.

## References

[1] Yao J.M., Wang L., Yan Y.Y., He F.L., Jian L. (2017). The supporting principle of truss cable in the rockburst roadway and its application. J. Min. Saf. Eng..

[2] Cao D.F., Zhang X., Lu Y.H., Wang K., Li C.Q., Liu Q., Han Z.X. (2021). Seismic performance of steel-truss reinforced concrete portal frame structures. J. Build. Struct..

[3] Yang J.J., Yao J., Peng H.K., Gu Z.Y., Wang Z.G. (2015). Research on rapid design method of spacecraft truss structure. Electro-Mech. Eng..

[4] Hu H.Y., Tian Q., Zhang W., Jin D.P., Hu G.K., Song Y.P. (2013). Nonlinear dynamics and control of large deployable space structures composed of trusses and meshes. Adv. Mech..

[5] Qiao D., Zhang L., Long P.P. (2014). Rapid splicing portable truss bridge technology research and application. Mech. Manag. Dev..

[6] Luo Y.X., Che X.Y., Liu Q.Y., Wang C. (2008). The research of hyper-chaotic neural network method to displacement analysis of variable geometry truss manipulator. J. Hunan Univ. Sci. Tech. (Nat. Sci. Edit.).

[7] Guan K., Pan C.Y., Zhang X., Deng H. (2010). Establishment and analysis of general kinematic model of plane scissor mechanism. J. Mech. Des. Res..

[8] Wu G.R. (2013). Study on Design Theory of Curve Trajectory Scissors. Master’s Thesis.

[9] Li J.L., Liu J.X., Wang Y.F. (2016). Development and finite element analysis of a scissor-type telescopic stent. Mach. Manuf..

[10] Han B., Xu Y.D., Yao J.T., Zhang S., Zhen D., Zhao Y.S. (2019). Design and analysis of an overconstrained scissors double-hoop truss deployable antenna mechanism. J. Yanshan Univ..

[11] Wu N., Liu R.Q., Guo H.W. (2014). The Design and Kinematics Analysis of an Underactuated Cable-Truss Mechanism. Adv. Mater. Res..

[12] Liu G., Chen W., Wang W., Chen Y. (2018). Design and analysis of a novel space deployable mechanism of ring and frustum type. Int. J. Adv. Manuf. Technol..

[13] Dai J.S., Li D., Zhang Q., Jin G. (2004). Mobility analysis of a complex structured ball based on mechanism decomposition and equivalent screw system analysis. Mech. Mach. Theory.

[14] Huang W.L. (2011). Research of Static Behavior of Tubular Gap KK-Joints Made of Square Chord and Circular Braces. Master’s Thesis.

[15] Li B., Cui Q.F. (2011). Topological characteristics of a spatial truss deployable mechanism. Mech. Sci. Tech. Aeros. Eng..

[16] Huang Z.B., Liu J.Y., Yuan T.T., Hou P. (2021). Dynamic modeling and analysis on a deployable space truss multi-body system. J. Vib. Shock.

[17] Yan Z.T., Cao J.Y., Zhu C.H., Zhai Y.Q. (2020). A new topology optimization structure of aluminum alloy truss. Sichuan Build. Sci..

[18] Chen J.J., Cao Y.B., Sun H.A. (2000). Topology optimization of truss structures with systematic reliability constraints under multiple load cases. Acta Mech. Solida Sin..

[19] Zhang L., Bhatti M.M., Marin M., Mekheimer K.S. (2020). Entropy Analysis on the Blood Flow through Anisotropically Tapered Arteries Filled with Magnetic Zinc-Oxide (ZnO) Nanoparticles. Entropy.

[20] Sun X., Jing X., Xu J., Cheng L. (2014). Vibration isolation via a scissor-like structured platform. J. Sound Vib..

[21] Chen C.Z., Wei J., Chen J.B., Nie H., Zheng G., Yuan Y.N. (2018). Characteristics analysis of landing buffer in the truss type mars lander. J. Vib. Meas. Diagn..

[22] Li Y.N., Zhang W., Cao D.X., Cui Y.T. (2017). Modal experiment analysis and FEM simulation for a ring truss structure. Chin. J. Appl. Mech..

[23] Marin M., Othman M.I.A., Seadawy A.R., Carstea C. (2020). A domain of influence in the Moore–Gibson–Thompson theory of dipolar bodies. J. Taibah Univ. Sci..

[24] Jamal-Omidi M., Benis A.C. (2021). A numerical study on energy absorption capability of lateral corrugated composite tube under axial crushing. Int. J. Crashworthiness.

[25] Li J., Tian C., Hong W., Duan S., Zhang Y., Wu W., Hu G., Xia R. (2021). Shock responses of nanoporous gold subjected to dynamic loadings: Energy absorption. Int. J. Mech. Sci..

[26] Yuan Z.Z., Dai Q.X. (2019). Metallic Materials.

[27] Dewey H.H., Pierce G.A. (2011). Introduction to Structural Dynamics and Aeroelasticity.

[28] Ministry of Housing and Urban-Rural Development of the People’s Republic of China (2001). Quality Standard of Steel Trusses (JG 8-1999).

[29] Zheng Y. (2017). Optimization Design of a Parallel Mechanism Driven by Nine Cables Based on Experimental Design Methods and Response Surface Methods. Chin. J. Mech. Eng..

[30] Besharatlou S., Anbia M., Salehi S. (2021). Optimization of sulfate removal from aqueous media by surfactant-modified layered double hydroxide using response surface methodology. Mater. Chem. Phys..

[31] Chen X.Y. (2007). Study on Crashworthiness Optimization of Thin-Walled Structure and Truss Structure. Master’s Thesis.

